# The Possible Role of TLR2 in Chronic Hepatitis B Patients with Precore Mutation

**DOI:** 10.1155/2013/780319

**Published:** 2013-09-25

**Authors:** Malihe Moradzadeh, Sirous Tayebi, Hossein Poustchi, Kourosh Sayehmiri, Parisa Shahnazari, Elnaz Naderi, Ghodratollah Montazeri, Ashraf Mohamadkhani

**Affiliations:** ^1^Department of Modern Sciences and Technologies, School of Medicine, Mashhad University of Medical Sciences, Mashhad, Iran; ^2^Liver and Pancreatobiliary Diseases Research Center, Digestive Diseases Research Institute, Tehran University of Medical Sciences, Tehran, Iran; ^3^Psychosocial Injuries Research Centre, Ilam University of Medical Sciences, Ilam, Iran; ^4^Monoclonal Antibody Research Centre, Avicenna Research Institute, ACECR, Tehran, Iran

## Abstract

Recognition mechanisms of innate immune response help to improve immunotherapeutic strategies in HBeAg-negative chronic hepatitis B (CHB). Toll-like receptor 2 (TLR2) is an important component of innate immunity. In this study, the frequency of precore mutations of the hepatitis B virus (HBV) and serum TLR2 were evaluated in CHB patients. Fifty-one patients with chronic hepatitis B, negative for HBeAg and detectable HBV DNA, were examined for the presence of mutations in pre-core region of HBV genome by direct sequencing. Serum TLR2 was measured by enzyme-linked immunosorbent assay. Interactions of truncated HBeAg and TLR2 proteins were evaluated with molecular docking software. The G1896A pre-core mutation were detected in 29 (57%) which was significantly associated with higher concentration of serum TLR2 in comparison with patients without this mutation (4.8 ± 2.9 versus 3.4 ± 2.2 ng/mL, *P* = 0.03). There was also a significant correlation between serum ALT and TLR-2 (*r* = 0.46; *P* = 0.01). Docking results illustrated residues within the N-terminus of truncated HBeAg and TLR2, which might facilitate the interaction of these proteins. These findings showed the dominance of G1896A pre-core mutation of HBV variants in this community which was correlated with serum TLR2. Moreover TLR2 is critical for induction of inflammatory cytokines and therefore ALT elevation.

## 1. Introduction

Hepatitis B virus (HBV) infection is an important cause of chronic hepatitis, cirrhosis, and hepatocellular carcinoma (HCC) [1]. The transmission of HBV from infected mothers to neonates causes persistent infection [2]. Chronic infection of HBV is a global health problem. However, the prevalence and genotype distribution of HBV are different among the geographical areas [3]. The majority of chronic hepatitis B patients lose HBe antigen (HBeAg) and develop anti-HBe antibody, which is generally associated with a decrease in serum HBV DNA levels and a gradual accumulation of precore or core promoter mutations [4]. HBeAg-negative chronic hepatitis B is the predominant type of CHB in Mediterranean inhabitants [3]. Two types of precore and core promoter HBV mutations that reduce HBeAg formation are more frequent in regions where patients are predominantly infected with HBV genotype D [4, 5]. Infection with wild-type strains of HBV often induces mild symptoms and responds well to interferon alpha therapy, but patients infected with precore mutant variants may show clinical evidence of elevated or fluctuating ALT and HBV DNA [6]. The reason that precore negative mutants become predominant in some patients during chronic hepatitis B infection is not clear. However, the host immune system has a functional role in the selection of precore mutant strains of HBV, and their appearance might reflect immunological control of infection [7, 8].

Infected hepatocytes are eliminated by vigorous CD4+ and CD8+ T-cell responses, and those who have insufficient cellular immune response will persist chronically infected [9]. The impact of innate immunity in liver damage also has been identified in several studies [10, 11]. Toll-like receptors (TLRs) describe a group of pattern recognition receptors (PRRs) playing critical roles in the host innate immune response [12]. These proteins are evolutionarily conserved from *Drosophila* to humans and important in controlling the activation of the adaptive immune response [13]. Various TLRs exhibit different patterns of expression [14]. Overactivation of TLRs plays a prominent role in the pathogenesis of a variety of acute and chronic inflammatory conditions [13]. A previous study reports that the HBeAg downregulates antiviral defenses of the host [15] and, in the absence of HBeAg, HBV replication is associated with upregulation of the TLR2 pathway, resulting in increased TNF-*α* production [16–19]. A wide range of microbial and viral components as well as several endogenous TLR ligands are recognized by TLR2 [14]. This receptor is expressed in peripheral blood leukocytes, mainly in monocytes, in lymph nodes, bone marrow, and spleen [20]. TLR2 is also released by normal monocytes and is present in serum and other biological fluids which mostly contain the TLR2 extracellular domain [20, 21].

The importance of diverse TLRs for the “*in vivo*” replication and pathogenesis of HBV have been evidenced in several reports [16, 17]. However, there are no reports of serum TLR2 in HBeAg negative chronic hepatitis B. In this study, the association of serum TLR2 with clinical findings in chronic hepatitis B patients especially in patients with G1896A stop codon mutation has been investigated.

## 2. Patients and Methods

### 2.1. Patients

A total of 51 chronic HBeAg negative patients with detectable HBV DNA and a range of normal to elevated ALT were evaluated during a period of 12 months. They perinatally acquired chronic infection as they had a clear history of familial HBV infection, without coinfection of human immunodeficiency virus (HIV), autoimmune hepatitis, and other hepatitis viruses. Blood samples were taken at the initial assessment before liver biopsy. Serum samples were stored at −70°C. No patient received anti-HBV therapy prior to liver biopsy. The protocol for the study was approved by the ethics committee of Shariati Hospital, Tehran, University of medical science.

### 2.2. Clinical Evaluation and HBV-DNA Quantification

The presence of HBsAg, HBeAg, anti-HBeAg, anti-HCV, anti-HDV, and anti-HIV were determined with commercial assay kit EIA, Dia.Pro diagnostic, Italy. Serum TLR2 was measured by toll-like Receptor2 ELISA Kit (Uscn Life Science, Wuhan, China) according to the manufacturer's specifications. Sensitivity of the ELISA for TLR2 was 0.312–20 ng/mL.

HBV DNA was extracted from 200 *μ*L of serum using QIAamp DNA Blood Mini Kit (QIAGEN, USA), eluted in 50 *μ*L of elution buffer, and then measured in the Light-Cycler (Roche) by RealARTTM HBV LC PCR (QIAGEN, Hilden, Germany) according to the manufacturer's instructions. This assay had a linear range of 10^2^–10^9^ copies/mL. Liver biopsies from all patients were assessed for the grade of histological activity and stage of fibrosis using the modified histological activity index (HAI) scoring system [22].

### 2.3. Precore G1896A Mutation Detection and Direct Sequencing

The precore region was analyzed by hemi-nested PCR according to Gan et al. [23]. The sense primer PC5 5′-TCG CAT GGA GAC CAC CGT GA-3′ (nt. 204–223) and the antisense primer PC2 5′-GGC AAA AAC GAG AGT AAC TC-3′ (nt. 540–559) were performed as first round primers. An additional hemi-nested round of amplification was achieved using 2 *μ*L of the first round product as template and the antisense primer 527 5′-GTA ACT CCA CAG WAG CTC C-3′ (nt. 528–546). The numbering organization for primer nucleotides was in accordance to the genome sequence HPBADR1CG [23]. The PCR products were purified using PCR purification kit from MO BIO Laboratories Inc. (Carlsbad, CA, USA) based on the manufacturer's instructions and eluted in 100 *μ*L of elution buffer. The sequencing of PCR products was accomplished by the Big Dye Terminator Cycle sequencing Ready Reaction Kit Version 3.1 (Applied Biosystems, Foster City, CA, USA).

### 2.4. Statistical Analysis

Normality of data was assessed using One Sample Kolmogorov-Smirnov Test. Correlations between variables were analyzed using Pearson correlation coefficient (*r*). The independent samples *t*-test is used to compare the means ± standard deviation (SD) of data. The Mann-Whitney *U* test was utilized to test equality of TLR2 and ALT between patients with G1896A precore mutation and patients without mutation. A *P* value <0.05 was deemed statistically significant.

### 2.5. HBeAg and TLR2 Interaction Analysis

In order to identify the strongly associated functional of HBeAg, the protein-protein interaction solutions were mapped between HBeAg and TLR2. The sequence of truncated HBeAg amino acid, created as a result of a stop codon at position 28 of HBeAg and genomic mutation at base 1898 of HBV, was extracted from UniProt (P0C6H9). The tertiary structure of truncated HBeAg was built by Pepstr [24]. The Pepstr server predicts the tertiary structure of small peptides with sequence length varying of 7 to 25 (residues http://www.imtech.res.in/raghava/pepstr/). The X-ray crystal structure of the TLR2 (2Z80A) was retrieved from PDB (Protein Data Bank) [25]. The *PatchDock* web server and a refinement by *FireDock* evaluate the molecular docking of both proteins [26, 27].

## 3. Results

### 3.1. Demographic and Clinical Characteristics of the Patients

Demographic characteristics and frequency of the G1896A precore mutation along with the clinical and biochemical profiles of study subjects are summarized in Table 1. There were a total of 51 patients (mean age 37 ± 10 yr) including 16 females and 35 males. The quantification of HBV DNA was reported in log copies/mL with a mean value of 3.46 ± 1.06 and 29 (57%) patients that showed the G1896A precore mutation. Total score of necroinflammatory grade and fibrosis stage were measured based on the modified HAI system and serum ALT were 4.8 ± 2.3 and 57 ± 56 IU/I, respectively. The mean concentration of serum TLR2 was 4.2 ± 2.7 ng/mL.

### 3.2. Clinical Significance of G1896A Precore Mutation and Serum TLR2

**Figure 1:**
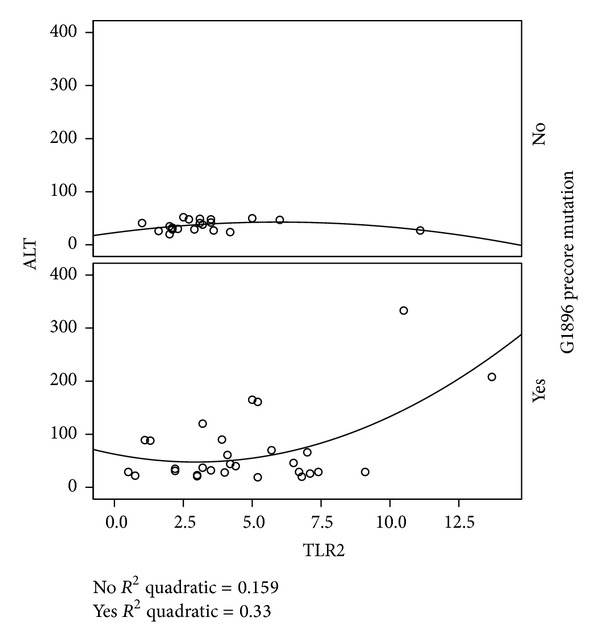
Estimating regression quadratic equation indicates that ALT sharply rises with increasing TLR2.

The concentration of serum TLR2 was higher in G1896A precore mutants than wild-type infected patients (4.8 ± 2.9 versus 3.4 ± 2.2 ng/mL, *P* = 0.032) (Table 1). There was no significant relationship between G1896A mutation variants with neither age nor sex. Patients infected with the wild-type HBV revealed similar mean of viral load compared to G1896A precore mutant patients (3.54 ± 1.02 versus 3.41 ± 1.1 log copies/mL). More likely, patients who harbored the precore mutant strains had higher levels of serum ALT and HIA score compared to those patients infected with wild-type strain of HBV, however it was not statistically significant (68 ± 67 versus 41 ± 27 IU/I and 5.3 ± 2.6 versus 4.2 ± 1.7) (Table 1). Serum TLR2 and ALT had significant correlation (*r* = 0.46; *P* = 0.01) which was more pronounced in patients with G1896A mutation (*r* = 0.48; *P* = 0.008) (Figure 1). Furthermore, there was statistically significant association between the log HBV DNA with serum ALT and with HIA score (*r* = 0.36; *P* = 0.09 and *r* = 0.3; *P* = 0.03). Serum ALT and HBV DNA did not show significant relationship to age, sex, and total grade of histological activity and stage of fibrosis.

### 3.3. TLR2 and Truncated HBeAg Interaction

**Figure 2:**
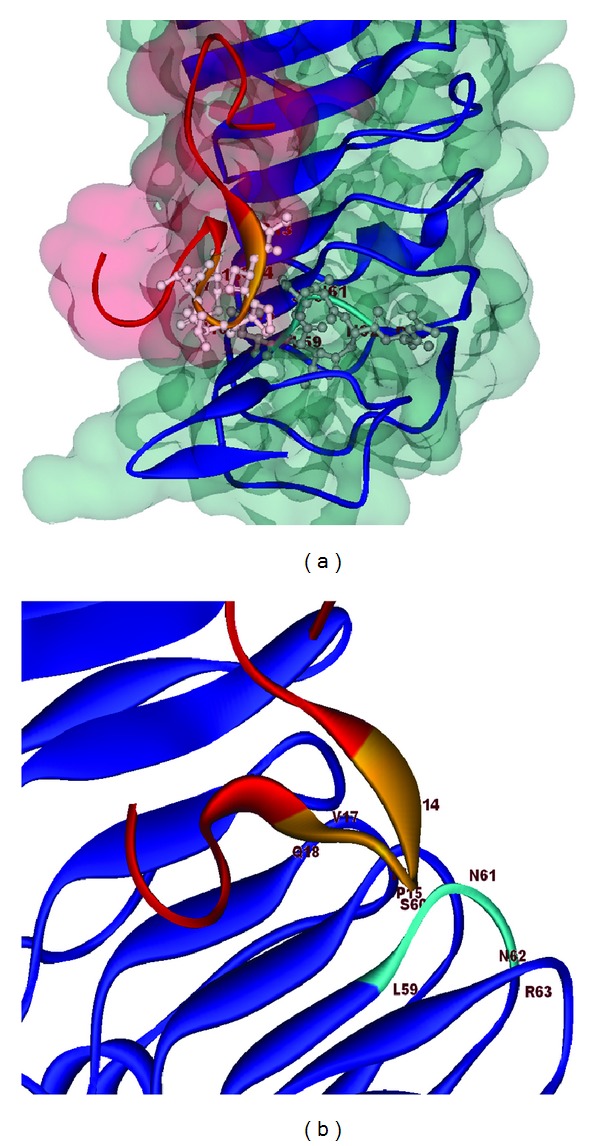
The docking result of HBeAg and TLR2. (a) The interaction of HBeAg with the accessible area of TLR2. (b) Docking of the a helices in the major groove of TLR2 with HBeAg as the ribbon form. The amino acids residues Cys14, Pro15, Thr16, Val17, and Gln18 of precore protein (HBeAg) colored in yellow bonding to Leu59, Ser60, Asn61, Asn62 and Arg63 of TLR2 that appeared in light blue.

In this experiment, the protein-protein docking of HBeAg and TLR2 were studied by PatchDock bioinformatic docking tool. Then, high-throughput refinement of docking was selected by FireDock (Figure 2). Protein-protein docking and interaction simulations disclosed hydrogen and ionic bonds. The amino acid residues Cys14, Pro15, Thr16, Val17 and Gln18 from HBVD-truncated HBeAg bonded to Asp58, Leu59, Ser60, Asn61, Asn62 and Arg63 of TLR2, respectively.

## 4. Discussion

The dynamic state of chronic HBV infection is a result of interactions between the virus itself and the host immune response [3]. Accordingly, different phases in the natural course of HBV infection are observed. Patients in immune tolerant phase usually have high viral load and normal levels of serum ALT. However, during the immune clearance phase, patients have moderate levels of HBV replication and elevated level of ALT [3, 28]. The HBeAg has been proposed as a viral approach to induce immunotolerance. The precore and basic core promoter (BCP) genetic variations of HBV lead to HBeAg loss and anti-HBe seroconversion [4, 29]. Principally, the stop codon mutation at base 1896 creates the truncated precore peptide that might represents an adaptation to immune pressure [30]. The function of innate immune response as the first line of defense against virus infection as well as its cooperation with adaptive immunity may induce the development of precore mutant variants of HBV [11, 31, 32]. Therefore, the clinical significance of these mutations appears to be linked to the function of host immune system.

The result of this study revealed the presence of HBV variants with precore mutations in 57% of anti-HBe-positive patients that explained the lack of HBeAg synthesis. Patients with precore mutants had higher concentrations of TLR2 compared to patients infected with wild-type HBV. Furthermore, increasing TLR2 is associated with serum ALT concentration in G1896A precore mutant patients. The increase of serum ALT could reflect the hepatitis activity and the host immune response against HBV that induces apoptosis and necrosis [3]. These findings are consistent with a previous study reporting the association of HBV replication and activation of TLR2 in precore mutant patients [17]. The members of TLRs family play an essential role in the innate immune recognition [12, 16], are upregulated in response to microbial components [33, 34], and are critical for the development of effective immunity [14]. A downregulation of TLR2 receptor in peripheral blood monocytes of chronic patients infected with HBeAg positive variants has been shown previously [17]. Visvanathan et al. reported that wild-type (HBeAg-positive) and the precore stop codon (HBeAg-negative) HBV variants had different effects on TNF-*α* production. Moreover, they showed that the expression of TLR2 on hepatocytes, Kupffer cells, and peripheral monocytes were significantly increased in HBeAg-negative chronic hepatitis B [17]. Lian et al. also found that the expression of TLR2 was significantly upregulated in patients with liver cirrhosis and chronic hepatitis B patients [9]. Interestingly, the therapeutic TLR strategy by Isogawa et al. revealed that TLRs ligands except for TLR2 are able to induce antiviral cytokines (Interferon *α*/*β*) at the site of HBV replication [16]. We therefore speculate that the increased serum TLR2 levels in our patients may correspond to higher expression of cellular TLR2 and consequently elevated TLR2 signaling leading to expression of proinflammatory cytokines that explain the chronicity of HBV infection that usually accompanies an increase in ALT.

In conclusion, our data show that the concentration of serum TLR2 was significantly higher in HBeAg negative patients with G1896A mutation which in turn was associated with higher serum ALT in this group. These results indicate the interaction of precore mutant strain of HBV and TLR2. Additional studies with longitudinal follow-up of subjects are required to determine the precise impact of TLR2 in HBeAg negative chronic hepatitis B.

## Figures and Tables

**Table 1:** Clinical and pathological data of 51 chronic hepatitis B patients with and without precore mutation G1896A.

Clinical factor*	All subjects (*n* = 51)	Patients with wild-type variant (*n* = 22)	Patients with G1896A mutation (*n* = 29)	*P* value
Age (years)	37 ± 10	36 ± 9	38 ± 11	0.4
log⁡HBV DNA (copies/mL)	3.46 ± 1.06	3.54 ± 1.03	3.41 ± 1.10	0.6
ALT (IU/L)	57 ± 56	41 ± 27	68 ± 67	0.5
TLR2 (ng/mL)	4.2 ± 2.7	3.4 ± 2.2	4.8 ± 2.9	0.03**
Histological activity index (HAI) score	4.8 ± 2.3	4.2 ± 1.7	5.3 ± 2.6	0.076

*Mean ± SD, **P value computed using the Mann-Whitney test.
